# Differentially private release of medical microdata: an efficient and practical approach for preserving informative attribute values

**DOI:** 10.1186/s12911-020-01171-5

**Published:** 2020-07-08

**Authors:** Hyukki Lee, Yon Dohn Chung

**Affiliations:** Department of Computer Science and Engineering, Korea University, 145 Anam-ro, Seongbuk-gu, Seoul, 02841 Republic of Korea

**Keywords:** Medical privacy, Data release, Data anonymization, Differential privacy, Privacy-preserving data publishing

## Abstract

**Background:**

Various methods based on *k*-anonymity have been proposed for publishing medical data while preserving privacy. However, the *k*-anonymity property assumes that adversaries possess fixed background knowledge. Although differential privacy overcomes this limitation, it is specialized for aggregated results. Thus, it is difficult to obtain high-quality microdata. To address this issue, we propose a differentially private medical microdata release method featuring high utility.

**Methods:**

We propose a method of anonymizing medical data under differential privacy. To improve data utility, especially by preserving informative attribute values, the proposed method adopts three data perturbation approaches: (1) generalization, (2) suppression, and (3) insertion. The proposed method produces an anonymized dataset that is nearly optimal with regard to utility, while preserving privacy.

**Results:**

The proposed method achieves lower information loss than existing methods. Based on a real-world case study, we prove that the results of data analyses using the original dataset and those obtained using a dataset anonymized via the proposed method are considerably similar.

**Conclusions:**

We propose a novel differentially private anonymization method that preserves informative values for the release of medical data. Through experiments, we show that the utility of medical data that has been anonymized via the proposed method is significantly better than that of existing methods.

## Background

### Introduction

In the last few decades, significant volumes of medical data have been collected and stored; consequently, there have been developments in the ability to process these data. Analytics on such stored data can help realize efficient healthcare services. For instance, data mining techniques applied to medical and social media data enable disease monitoring as well as health-based trend analyses. Furthermore, analyzing data of varying natures can help acquire new knowledge and intelligence, explore new hypotheses, and identify hidden patterns [1, 2].

Although possessing medical data benefits the data holders, it is occasionally necessary to release these data. For example, if data holders are not experts in conducting data analyses, they should outsource such analyses to a third-party. However, privacy concerns must take precedence during such a release of data, because the data might include sensitive information, such as the disease statuses of individuals. Several privacy models have been proposed to protect the privacy of individuals. These models can be broadly categorized into two types: (1) *k*-anonymity and its extensions [3–6] and (2) differential privacy [7].

The concept of *k*-anonymity was introduced by Sweeney and Samarati [3]. In this model, each record of an individual contained in a released dataset cannot be distinguished from the records of at least *k*-1 other individuals. *k*-anonymity can reduce the risk of privacy breaches under certain assumptions; however, various studies have indicated the vulnerability of *k*-anonymity and proposed stronger privacy models such as *l*-diversity, *t*-closeness, and *p*-sensitive [4–6]. These privacy models are similar to *k*-anonymity as they guarantee privacy through syntactic conditions; thus, they are termed *syntactic privacy models*. Although syntactic privacy models can effectively protect privacy under certain conditions, they are inherently vulnerable to various attacks [8].

In contrast to syntactic privacy, differential privacy (also known as semantic privacy) provides a more rigorous guarantee of privacy, regardless of the background knowledge of adversaries. Dwork et al. introduced the concept of *ε*-differential privacy [7], which provides a mathematically provable guarantee of protecting the privacy of individuals. The goal of differential privacy is that the output of a query should not be considerably influenced when a single record is added or removed. Differential privacy has emerged as the *de-facto standard* for privacy-preserving data analyses.

Differential privacy typically targets privacy-preserving data mining, which responds to query processing of the data rather than the publishing of microdata. Although some methods for publishing differentially private data based on non-interactive settings have been proposed, these methods focus on aggregated results such as histograms or contingency tables [9, 10]. However, if the domain of informative attributes used for the analysis is large, such as the disease attributes in medical data, it is difficult to create a contingency table. In several real-world data publishing scenarios, releasing microdata is even more suitable due to the flexibility it yields to data analysts. Consequently, in this paper, we propose a method called ***IPA*** (Informative attribute Preserving Anonymization) for publishing medical microdata under differential privacy. This study focuses on the method to perturb a raw dataset to provide differentially private results on a record-by-record basis, while improving data utility by preserving informative attributes.

### Motivation

The most commonly used method to achieve differential privacy is the addition of noise to the results. In a previously reported approach, noise was added to a contingency table of the raw dataset under non-interactive settings [9]. This implies that noise is added to every possible combination of the domain values for all attributes, irrespective of the existence of a record that corresponds to each combination in the raw dataset. For instance, suppose that we prepare a differentially private contingency table for the raw medical dataset listed in Table 1. The records are aggregated using all attributes, i.e., *Age, Gender and Disease*, to create a contingency table, which is presented as Table 2. Thereafter, noisy counts are added to every possible combination of the domain values for each attribute to achieve differential privacy, as shown in Table 3. If the dataset features many dimensions and/or the dimensions have large domains, a large amount of noise should be added. This leads to extreme distortion in the data.

**Table 1:** Original table

Age	Gender	Disease
10	M	Anemia
14	F	Gastritis
19	F	Pneumonia
12	F	Anemia
15	M	Pneumonia

**Table 2:** Contingency table created using Table 1

Age	Gender	Disease	Count
10	M	Anemia	1
12	F	Anemia	1
14	F	Gastritis	1
15	M	Pneumonia	1
19	F	Pneumonia	1

**Table 3:** Noisy version of contingency table

Age	Gender	Disease	Noisy count
10	M	Anemia	2
10	M	Gastritis	0
10	M	Pneumonia	1
10	F	Anemia	0
...	...	...	...
19	F	Gastritis	1
19	F	Pneumonia	1

To reduce the information loss caused by noise, generalization-based approaches have been proposed [10]. These approaches generalize original data by converting raw domain values with more general but semantically consistent values; for example, a specific *Age* value of 13 can be generalized into the interval [10-19 ]. Table 4 presents an example of a generalized contingency table. All the records have been generalized into indistinguishable groups, which are called *equivalent classes*, such as <[10-19 ], ∗, and Anemia >. Due to this generalization, the number of combinations is reduced; consequently, the total number of noisy counts is decreased.

**Table 4:** Generalized noisy version of contingency table

Age	Gender	Disease	Noisy count
[10−19]	*	Anemia	3
[10−19]	*	Gastritis	1
[10−19]	*	Pneumonia	1

It should be noted that generalization also distorts data, although the amount of distortion is less than that caused by noise. In particular, when informative attributes are generalized, the quality of data is affected considerably. Previous methods limit the informative attributes used for analyses to *Class* attributes (i.e., True or False) and do not generalize informative attributes. Therefore, it is difficult to use these methods for publishing medical data, because such data typically involve informative attributes with large domains, such as those of diseases and medications. In this study, we neither generalize the informative attributes nor do we create contingency tables; instead, we publish anonymized microdata with raw informative values.

### Contributions

Although several methods for releasing anonymized data have been proposed, a majority of these methods are based on syntactic privacy models [11, 12]. As mentioned above, stronger guarantees of privacy through differential privacy are required to protect the privacy of an individual. Furthermore, some of the previous works on publishing differentially private data are only relevant for classification analyses [13, 14]. In this paper, we propose a data anonymization method based on the differential privacy theory. To the best of our knowledge, this is the first work to propose a differentially private microdata publishing method for informative attributes with large domains. We evaluate the performance of the proposed method in terms of data utility and accuracy, through real-world analyses. The contributions of this study are as follows:

- We design a data anonymization method in which informative attributes remain unperturbed, while still complying with differential privacy. Regardless of the type and domain of the attribute, the raw informative values are preserved.
- We devise an algorithm that identifies useful anonymized datasets. This algorithm provides differentially private and high-utility anonymized datasets.
- We conduct extensive experiments and compare the proposed method with related existing methods. The experimental results prove that the proposed algorithm significantly improves data utility and also provides a rigorous privacy guarantee.

### Preliminaries

Differential privacy is a rigorous privacy model that does not involve any assumptions regarding the background knowledge of adversaries. It guarantees that almost no difference will be observed in the output of any query when a single record is added to or removed from the database. Formally, differential privacy is defined as follows:

#### Definition 1.

(*ε*-differential privacy) Assume a mechanism $\mathcal {A}$ that randomizes query outputs and any pair of neighboring databases $\mathcal {D}$ and $\mathcal {D}^{\prime }$. Then, $\mathcal {A}$ satisfies *ε*-differential privacy if and only if:

1 $$\begin{array}{*{20}l} &Pr\left[\mathcal{A}\left(\mathcal{D}\right)=S\right] \leq exp\left(\epsilon \right) \times Pr\left[\mathcal{A}\left(\mathcal{D}^{\prime}\right)=S\right]\\&\quad \text{where}~ \mathcal{S} \in Range(\mathcal{A}).  \end{array} $$

□

We assume that $\mathcal {D}$ and $\mathcal {D}^{\prime }$ are neighboring databases if they differ in exactly one record. In particular, we can obtain $\mathcal {D}^{\prime }$ from $\mathcal {D}$ by adding or removing an arbitrary record. If Eq. 1 is satisfied, there is a high probability that $\mathcal {D}$ and $\mathcal {D}^{\prime }$ produce the same query results. Therefore, even an adversary with maximal background knowledge cannot infer a particular record.

#### Definition 2.

(Sensitivity) For all $\mathcal {D}$ and $\mathcal {D}^{\prime }$, the sensitivity of the function *f* is defined as

2 $$\begin{array}{*{20}l} \Delta f=\max_{\mathcal{D},\mathcal{D}^{\prime}}\left|\left|f\left(\mathcal{D}\right)-f\left(\mathcal{D}^{\prime}\right)\right|\right|. \end{array} $$

□

Sensitivity is the maximal change inflicted on the output, when adding or removing an arbitrary record. Assume that the function *f* answers count queries over a dataset $\mathcal {D}$. Then, for any neighboring dataset $\mathcal {D}^{\prime }$, the result from *f* would differ by at most 1; therefore, the sensitivity of *f* would be 1.

To satisfy differential privacy, two mechanisms have been proposed: the *Laplace mechanism* and the *exponential mechanism* [7, 15]. The Laplace mechanism adds noise to the output of the function; this noise is sampled from a Laplace distribution. The noise is decided based on the privacy parameter *ε* and the sensitivity of the function *Δf*.

#### Theorem 1.

(Laplace mechanism) Let *f*($\mathcal {D}$) denote an output from the database $\mathcal {D}$. The Laplace mechanism satisfies *ε*-differential privacy if the random noise sampled from the Laplace distribution with mean *μ*=0 and scale *b*= *Δf*/*ε* is added to *f*($\mathcal {D}$). □

The exponential mechanism is used with maximum utility when the output of the function is an object and not a real value. The aim of this exponential mechanism is to choose the output with the highest score. It assigns scores to possible outputs using a score function. Thereafter, the mechanism randomly selects an output from the possible result set. The likelihood of selection increases exponentially for the outputs with higher scores.

#### Theorem 2.

(Exponential mechanism) Let $\mathcal {R}$ be the possible results of the function *f*. For the score function $\mathcal {S}:\mathcal {D} \times \mathcal {R} \rightarrow \mathbb {R}$, a mechanism that outputs $r \in \mathcal {R}$ with a probability that is proportional to $exp\left (\frac {\epsilon \mathcal {S}(\mathcal {D},r)}{2 \Delta \mathcal {S}}\right)$ satisfies *ε*-differential privacy, where $\Delta \mathcal {S}$ is the sensitivity of $\mathcal {S}$. □

Differential privacy involves two composition properties: sequential composition and parallel composition [16]. Sequential composition is applicable to cases wherein a sequence of computations is performed on a single dataset, whereas parallel composition is applicable to a sequence of computations on disjoint datasets.

#### Theorem 3.

(Sequential composition) Let each function *f*_*i*_ provide *ε*_*i*_-differential privacy. Thus, sequentially running all functions *f*_*i*_ over the dataset $\mathcal {D}$ provides $\left (\sum _{i} \epsilon _{i}\right)$-differential privacy. □

#### Theorem 4.

(Parallel composition) Let each function *f*_*i*_ provide *ε*_*i*_-differential privacy. Thus, applying each function over a set of disjoint datasets $\mathcal {D}_{i}$ provides max*i*(*ε*_*i*_)-differential privacy. □

**Fig. 1:**
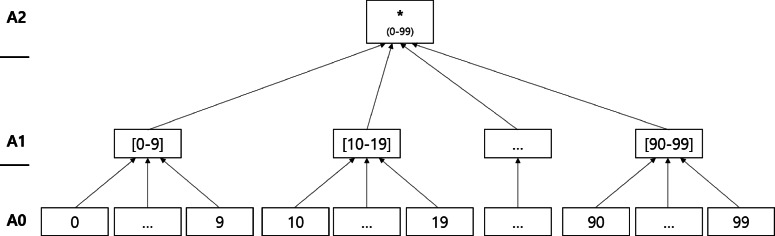
Taxonomy tree of the *Age* attribute

**Fig. 2:**
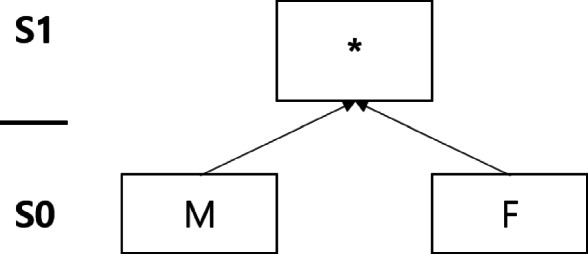
Taxonomy tree of the *Sex* attribute

**Fig. 3:**
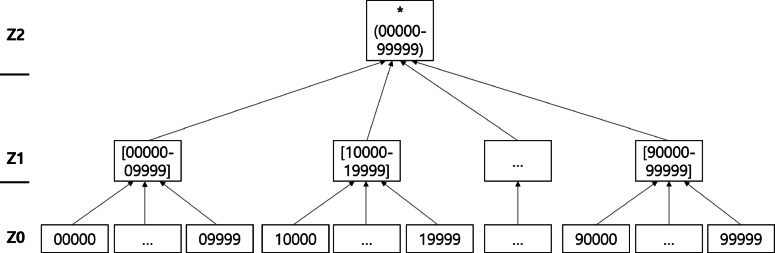
Taxonomy tree of the *Zip* attribute

Generalization refers to replacing original values with less specific values. Generalized values are specified by a predefined generalization hierarchy. Figures 1, 2, and 3 present taxonomy trees representing the generalization hierarchies of the attributes *Age*, *Sex*, and *Zip*, respectively. Suppression involves substituting a specific value from the original dataset with a special symbol such as “ ∗,” which denotes “anything” in the anonymized dataset. In Figures 1, 2, and 3, ∗ is the suppressed value.

**Fig. 4:**
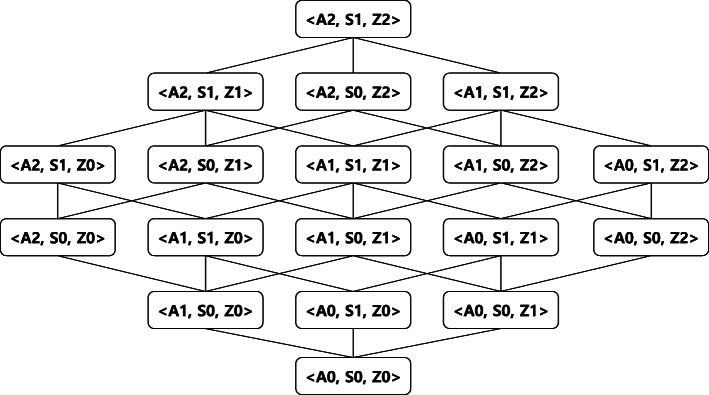
Generalization hierarchical lattice

When anonymizing datasets, we employ the *full-domain generalization* algorithm [17], which maps the entire domain of an attribute in the initial microdata to a more general domain, based on its domain generalization hierarchy (also known as its taxonomy tree). Taxonomy trees of the attributes are combined to form a multi-attribute generalization hierarchical lattice. Figure 4 depicts an example of such a generalization lattice. Each combination, such as <A1, S0, Z0 >, is called a *node*. The notation <A1, S0, Z0 > indicates that all values in the *Age* attribute have been generalized using A1 in the taxonomy tree ({[0−9],[10−19],...,[90−99]}) and that the *Sex* and *Zipcode* attributes have been generalized using S0 and Z0, respectively, (i.e., they are not generalized). The algorithm generalizes the dataset and measures information loss in the generalized dataset for each node of the lattice. The node with the lowest information loss is returned.

## Methods

### Problem settings

Consider that a data holder possesses a dataset $\mathcal {D}$ that contains multi-dimensional records, and each record belongs to a unique individual. This data holder wants to release an *ε*-differential private version of $\mathcal {D}$ with high data utility. It should be noted that all personal identifiable information, such as *SSNs (Social Security Numbers)*, has already been removed. $\mathcal {D}$ is defined as a set of records, and each record consists of a set of dimension attributes *A*_*dim*_= {*A*_1_,...,*A*_*q*_} belonging to individuals, such as their age and gender. The *A*_*dim*_ attribute values of an individual might be acquired via publicly available data sources such as those on the world wide web and social networking services; thus, adversaries could easily obtain these values. Additionally, $\mathcal {D}$ contains informative large-domain categorical attributes *A*_*inf*_ that are used for data analyses. The *A*_*inf*_ attribute values are private information, and adversaries cannot obtain these values. Privacy breaches occur if adversaries gain knowledge regarding the *A*_*inf*_ values. We assume that each attribute *A*_*i*_∈*A*_*dim*_ has a predefined taxonomy tree.

### Basic concepts

**Fig. 5:**
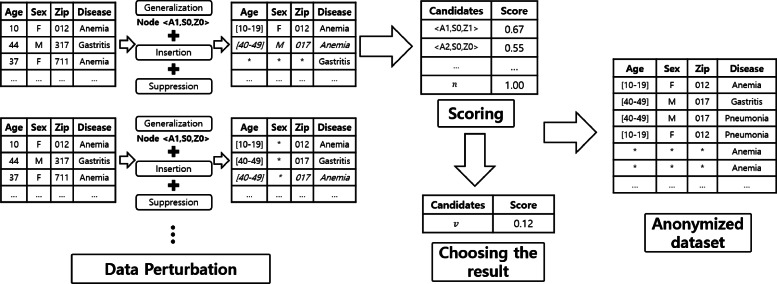
Process of *IPA*

In this section, we introduce the overall process of the proposed anonymization method (*IPA*). *IPA* consists of three steps: (1) generating candidates for data perturbation, (2) utility scoring of all candidates, and (3) choosing the result based on the scores. Figure 5 presents the process of *IPA*.

Data perturbation is essential for anonymization, and several data perturbation techniques are available. We adopt three data perturbation methods: *generalization, suppression*, and *insertion*; these methods were chosen for specific reasons. Noise insertion is a typical method of achieving differential privacy; however, the insertion-only approach involves substantial information loss due to the amount of noise. In terms of differential privacy, generalization does not help satisfy the privacy requirement. However, it can be used to improve utility by reducing noise and the domain size. Suppression is applied to equivalent classes containing few records. It helps reduce the number of counterfeit records; its details are described in subsequent sections. As *IPA* employs full-domain generalization, it generates candidates of perturbed datasets for all nodes in the generalization hierarchical lattice. Subsequently, the score of each dataset is measured based on the information loss and a result dataset is then selected. It should be noted that deterministic algorithms cannot satisfy differential privacy. Therefore, we employed the exponential mechanism to choose the node that will be the result dataset. In *IPA*, we allocate the privacy budget over four different parts, i.e., suppression threshold, number of counterfeit records, determining the informative attribute value of a counterfeit record, and choosing an anonymized dataset, which are proved by Theorems 5, 6, 7, and 8, respectively.

### Step 1: data perturbation

In *IPA*, all dimension attributes *A*_*dim*_= {*A*_1_,...,*A*_*q*_} are generalized using a predefined taxonomy tree (line 2 in Algorithm 1). The values of informative attributes (also known as measure attributes) remain unchanged during the generalization phase. The domain of generalized values is determined using the taxonomy tree. For example, Table 5 is an original table with *A*_*dim*_ = {*Age, Gender, Zipcode*} and *A*_*inf*_ = {*Disease*}. Table 6 presents a generalized version of Table 5. As a result of this generalization, the values of attributes *A*_1_,...,*A*_*q*_ in the same equivalent class become indistinguishable. This implies that the unit of the disjoint dataset has changed from a single record to an equivalent class. According to the parallel composition theorem, adding Laplace noise to each disjoint dataset can achieve differential privacy. Therefore, noise decreases as the number of equivalent classes decreases. When determining the *generalization* boundary, the privacy budget is not allocated. The generalization boundary is typically determined using the predefined taxonomy tree and not through a particular value or by distributing the dataset. Thus, one record does not affect the generalization boundaries of other records. Therefore, privacy breaches do not occur when determining the generalization boundary.

**Table 5:** Original table

Age	Gender	Zipcode	Disease
17	M	28912	Gastritis
16	M	23512	Pneumonia
13	M	24231	Pneumonia
24	F	31891	Anemia
29	F	34225	Anemia
25	F	37756	Diabetes
67	M	80061	Stroke

**Table 6:** Generalized table

Age	Gender	Zipcode	Disease
[10−19]	M	[20000−29999]	Gastritis
[10−19]	M	[20000−29999]	Pneumonia
[10−19]	M	[20000−29999]	Pneumonia
[20−29]	F	[30000−39999]	Anemia
[20−29]	F	[30000−39999]	Anemia
[20−29]	F	[30000−39999]	Diabetes
[60−69]	M	[80000−89999]	Stroke


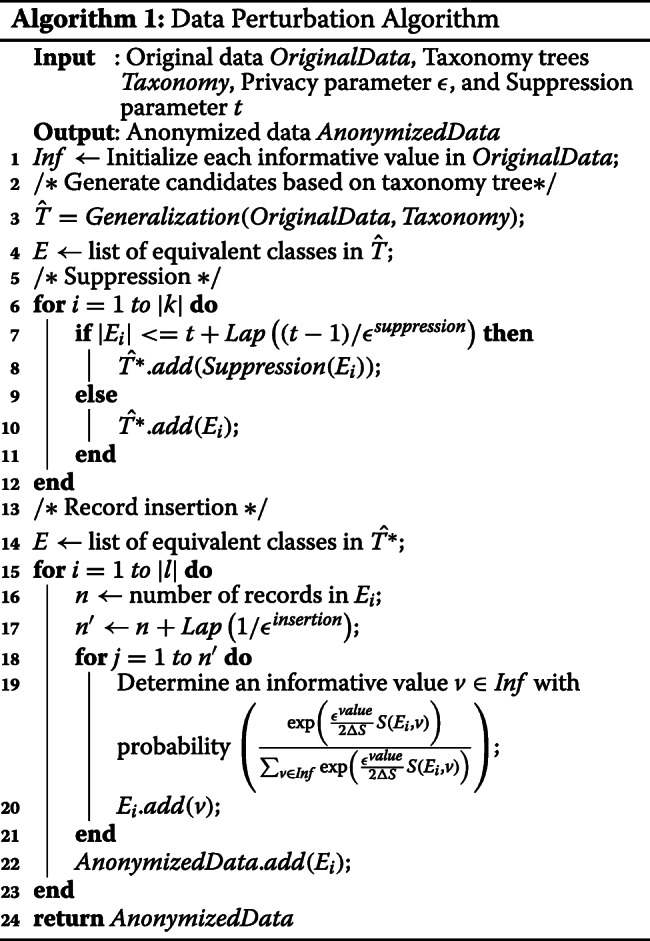


In full-domain generalization, a given value is mapped to a pre-determined generalized value (or interval) for all records. Accordingly, an adversary can realize that a specific record is not present in the original dataset if its corresponding equivalent class does not exist in the result dataset. To prevent this type of privacy breach, we adopt the suppression technique (lines 6-12 in Algorithm 1). Suppression implies that all dimension attribute values of a record are substituted with “ ∗,” which can be mapped to all the values in the domain. Because of the suppressed equivalent classes, adversaries will be unable to identify the equivalent class of the suppressed record. For example, in Tables 5 and 7, <[60-69], M, [80000-89999], Stroke > is suppressed to <∗, ∗, ∗, Stroke >. As the suppressed record is unknown, adversaries cannot identify the suppressed equivalent class from all other equivalent classes, except for the equivalent classes in the table. Furthermore, utility can also be improved via suppression. This is because suppression is performed on the generalized dataset and only a small amount of noise is added, as compared to the addition of noise for every possible equivalent class. We use the hyper-parameter *t* as the threshold for suppression. If the number of records in an equivalent class is less than or equal to *t*, the equivalent class is suppressed. For example, if we set *t* = 2, as the equivalent class corresponding to <[60-69], M, [80000-89999] > contains only one record, it is suppressed. All attribute values except the measure attributes are represented as “ ∗.” However, it should be noted that using a fixed threshold value can result in a privacy breach. Assume that there are exactly *t* records in an equivalent class. Thus, the inclusion or exclusion of one record determines whether or not the equivalent class is suppressed. Accordingly, *IPA* uses the Laplace mechanism to add noise to the threshold value. Let the threshold be *t* and the Laplace noise be *T*∼*Lap*((*t*−1)/*ε*^*suppression*^). Then, the noisy threshold is *t*+*Lap*((*t*−1)/*ε*^*suppression*^) (line 7), and sensitivity of the suppression threshold is (*t*−1). More formally, suppression is defined as follows:

**Table 7:** Suppressed table

Age	Gender	Zipcode	Disease
[10−19]	M	[20000−29999]	Gastritis
[10−19]	M	[20000−29999]	Pneumonia
[10−19]	M	[20000−29999]	Pneumonia
[20−29]	F	[30000−39999]	Anemia
[20−29]	F	[30000−39999]	Anemia
[20−29]	F	[30000−39999]	Diabetes
*	*	*	Stroke

#### Definition 3.

(Suppression) Let OT be the original table, GT be the generalized table, *t* be the suppression threshold, *ε*^*suppression*^ be the privacy budget, and *E*_*i*_(*i*=1,...,*k*) be an equivalent class in GT. If |*E*_*i*_|≤*t*+*Lap*((*t*−1)/*ε*^*suppression*^), *E*_*i*_ is suppressed. □

#### Theorem 5.

(Suppression threshold based on Definition 3 achieves (*ε*^*suppression*^)-differential privacy.)

#### *Proof*

Let (*t*−1) be the sensitivity of a suppression threshold. Thus, the privacy budget is *ε*^*suppression*^, and a differentially private version of the suppression threshold is *t* + *Lap*((*t*−1)/*ε*^*suppression*^). Based on Theorem 1, adding noise generated using the Laplace distribution *Lap*((*t*−1)/*ε*^*suppression*^) to the suppression threshold achieves (*ε*^*suppression*^)-differential privacy. □

To comply with differential privacy, counterfeit records are inserted into equivalent classes as noise (lines 14-23). Two aspects need to be considered when inserting these counterfeit records. First, the number of counterfeit records to be inserted into each equivalent class needs to be determined. We use the Laplace mechanism to determine the number of counterfeit records to be inserted. Let the number of records in an equivalent class be *n* and the Laplace noise be *C*∼*Lap*(1/*ε*^*insertion*^). Thus, the size of an equivalent class, excluding suppressed or empty records, is *n*+*Lap*(1/*ε*^*insertion*^) (lines 16-17).

#### Theorem 6.

(Inserting *n* + *Lap*(1/*ε*^*insertion*^) counterfeit records achieves (*ε*^*insertion*^)-differential privacy.)

#### *Proof*

Let the sensitivity of a count query be 1, privacy budget be *ε*^*insertion*^, and number of counterfeit records be *n* + *Lap*(1/*ε*^*insertion*^). All equivalent classes have exclusive boundaries determined using Theorems 1 and 4. Thus, adding independently generated counterfeit records from the Laplace distribution *Lap*(1/*ε*^*insertion*^) to each equivalent class achieves (*ε*^*insertion*^)-differential privacy. □

Thereafter, we need to determine the informative attribute values of newly inserted records. The smaller the distortion in the informative value ratio of an equivalent class, the better the utility. Therefore, in *IPA*, informative attribute values are determined using the exponential mechanism with the ratio of number of informative values in an equivalent class. Let *Count*_*i*_(*v*) be the number of records that have the informative value *v* in *E*_*i*_, where *E*_*i*_ is an equivalent class, *Inf* be a domain of informative values in *OriginalData*, and *Inf*_*i*_ be a domain of informative values in *E*_*i*_. |*E*_*i*_| denotes the number of records in *E*_*i*_, |*Inf*| denotes the size of *Inf*, and |*Inf*_*i*_| denotes the size of *Inf*_*i*_. The score function is calculated as follows:

3 $$\begin{array}{*{20}l} S(E_{i},v)= \left\{\begin{array}{ll} \frac{Count_{i}(v)}{\left|E_{i}\right|+1} & \text{if } v \text{ exists in } E_{i}\\ \frac{1}{\left(\left|E_{i}\right|+1\right)*\left(\left|Inf\right|-\left|Inf_{i}\right|\right)} & \text{otherwise} \end{array}\right.  \end{array} $$

Based on the scores of all candidates for the informative values, the exponential mechanism selects a candidate *v* with the following probability (line 19):

4 $$\begin{array}{*{20}l} \frac{\exp\left(\frac{\epsilon^{value}}{2\Delta S} S(E_{i},v)\right)} {\sum_{v \in Inf} \exp\left(\frac{\epsilon^{value}}{2\Delta S} S(E_{i},v)\right)}  \end{array} $$

An example is presented in Table 8. Two records have been inserted: <[10−19], *M*, [20000−29999], *Gastritis*> (Row 4) and <[20−29], *F*, [30000−39999], *Anemia*> (Row 8).

**Table 8:** Inserted table

Age	Gender	Zipcode	Disease
[10−19]	M	[20000−29999]	Gastritis
[10−19]	M	[20000−29999]	Pneumonia
[10−19]	M	[20000−29999]	Pneumonia
[10−19]	M	[20000−29999]	Gastritis
[20−29]	F	[30000−39999]	Anemia
[20−29]	F	[30000−39999]	Anemia
[20−29]	F	[30000−39999]	Diabetes
[20−29]	F	[30000−39999]	Anemia
*	*	*	Stroke

#### Theorem 7.

(Determining informative attribute values for inserted records based on Eq. 4 achieves (*ε*^*value*^)-differential privacy.)

#### *Proof*

Let *Inf* be the set of candidate values from which an informative attribute value is to be chosen. The *IPA* method selects a value *v* ∈*Inf* with the probability given in Eq. 4, where *S*(*E*_*i*_,*Inf*) is a score function and *ΔS* is the sensitivity of function *S*. Based on Theorem 2, choosing an informative value with a probability proportional to $\exp \left (\frac {\epsilon ^{value}}{2\Delta S}\right)$ satisfies (*ε*^*value*^)-differential privacy. □

### Step 2: scoring all candidates

We employ the information loss caused by data perturbation as a score function. In *IPA*, there are three factors that cause information loss.

The first factor is generalization. To measure the information loss caused by generalization, we introduce the concept of the NCP (Normalized Certainty Penalty) [18]. Let *v* be a value, |*v*| be the number of leaf nodes covered by *v* corresponding to the generalization hierarchy, and $|\mathcal {L}|$ be the total number of leaf nodes in the generalization hierarchy. Then, the NCP of a value is defined as follows:

5 $$\begin{array}{*{20}l} NCP_{value}(v)= \left\{\begin{array}{ll} 0, & |v|=1(v is leaf)\\ \frac{|v|}{|\mathcal{L}|}, & otherwise \end{array}\right. \end{array} $$

6 $$\begin{array}{*{20}l} NCP(\hat{\mathcal{D}})=\frac{\sum_{\forall r \in \hat{\mathcal{D}}}\sum_{\forall \mathcal{A}_{dim} \in \hat{\mathcal{D}}} NCP_{value}(v)}{|\hat{\mathcal{D}}|}  \end{array} $$

The value of NCP lies between 0 (i.e., minimum generalization) and 1 (i.e., maximum generalization). Therefore, the sensitivity of $\Delta NCP\left (\hat {\mathcal {D}}\right)$ is 1.

The second factor involves the distortion caused by inserted records. To measure this distortion, we employ the EMD (Earth Movers’s Distance) measure, which evaluates the dissimilarity between two multi-dimensional distributions [5]. For two distributions of the original and anonymized datasets, i.e., $P_{\mathcal {D}}=\left (p_{1},p_{2},...,p_{m}\right)$ and $Q_{\hat {\mathcal {D}}}=\left (q_{1},q_{2},...,q_{m}\right)$, respectively, the EMD is defined as follows:

7 $$\begin{array}{*{20}l} EMD\left[P_{\mathcal{D}},\ Q_{\hat{\mathcal{D}}}\right]=\frac{1}{2}\sum\limits^{m}_{k=1}\ \left|p_{k}-q_{k}\right|  \end{array} $$

The EMD of two completely different equivalent classes is at most 1. Thus, the sensitivity of the EMD $\Delta EMD\left [P_{\mathcal {D}},\ Q_{\hat {\mathcal {D}}}\right ]$ is 1.

Finally, the third factor in loss is the proportion of counterfeit records in equivalent classes, which can be defined as follows:

8 $$\begin{array}{*{20}l} Rate_{class}(E_{i}) = \frac{\left|Counterfeit_{i}\right|}{\left|E_{i}\right|} \end{array} $$

where *Counterfeit*_*i*_| denotes the number of counterfeit records inserted into *E*_*i*_, and the sensitivity *ΔRate*_*class*_(*E*_*i*_) is 1. *Rate* of the anonymized dataset $\hat {\mathcal {D}}$ is defined as follows:

9 $$\begin{array}{*{20}l} Rate(\hat{\mathcal{D}}) = \frac{\sum_{\forall E_{i} \in \hat{\mathcal{D}}} Rate_{class}(E_{i})}{The\ number\ of\ equivalent\ classes}  \end{array} $$

We use the sum of these three metrics to determine the total information loss.

10 $$\begin{array}{*{20}l} IL(\hat{\mathcal{D}}) = NCP\left(\hat{\mathcal{D}}\right) + EMD\left[P_{\mathcal{D}},\ Q_{\hat{\mathcal{D}}}\right] + Rate\left(\hat{\mathcal{D}}\right) \end{array} $$

As sensitivity of each metric is 1, the sensitivity of information loss $\Delta IL\left (\hat {\mathcal {D}}\right)$ is 3.

### Step 3: choosing the result

In this section, we discuss the method of choosing a result from the set of candidates. Furthermore, we prove that *IPA* is differentially private.

We first measure the score of all candidates and then choose a result. To assign a high score to the dataset with low information loss, the score function *u* is calculated as follows:

11 $$\begin{array}{*{20}l} u\left(\hat{\mathcal{D}}\right)=\left(3-IL\left(\hat{\mathcal{D}}\right)\right) \end{array} $$

Let *Candidates*_*i*_ be the set of candidate anonymized datasets; thus, the result is selected using probability:

12 $$\begin{array}{*{20}l} \frac{\exp\left(\frac{\epsilon^{candidates}}{2\Delta u} u\left(\hat{\mathcal{D}}\right)\right)} {\sum_{result \in Candidates_{i}} \exp\left(\frac{\epsilon^{candidates}}{2\Delta u} u\left(\hat{\mathcal{D}}\right)\right)}  \end{array} $$

Algorithm 2 illustrates the algorithm for choosing a result node. The algorithm begins with the creation of the hierarchical generalization lattice (line 1). Thereafter, the algorithm perturbs the original dataset for each node and calculates information loss (lines 2-5). After perturbing the dataset, a result is determined (line 7). The source code for Algorithms 1 and 2 is publicly available at GitHub [19].

#### Theorem 8.

(Choosing an anonymized dataset according to Algorithm 2 achieves (*ε*^*candidates*^)-differential privacy.)

#### *Proof*

Let *Candidates*_*i*_ be the set of candidate datasets from which a single anonymized dataset is chosen. *IPA* selects the dataset *result* ∈ *Candidates*_*i*_ using the probability in Eq. 12, where $u\left (\hat {\mathcal {D}}\right)$ is a score function and *Δu* is the sensitivity of the function *u*. Based on Theorem 2, choosing an informative value with a probability proportional to $\exp \left (\frac {\epsilon ^{candidates}}{2\Delta u}\right)$ achieves (*ε*^*candidates*^)-differential privacy. □


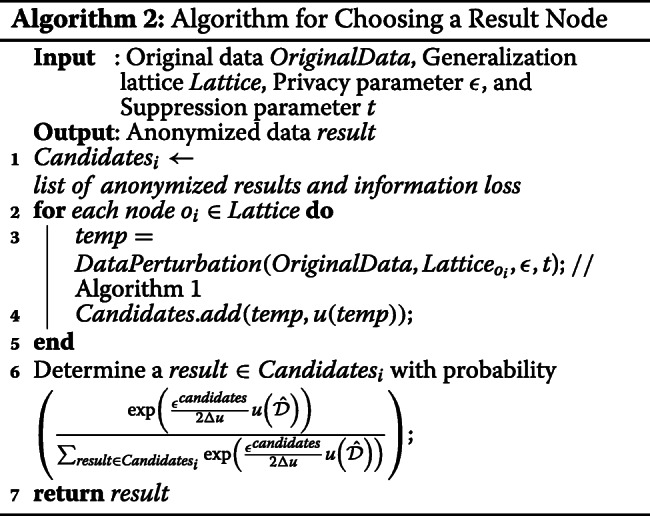


Thus, we have proven that each part of *IPA* guarantees differential privacy. These parts run on the same dataset; therefore, according to Theorem 3, *IPA* achieves (*ε*^*suppression*^+*ε*^*insertion*^+*ε*^*value*^+*ε*^*candidates*^)-differential privacy.

#### Theorem 9.

(*IPA* achieves (*ε*^*suppression*^+*ε*^*insertion*^+*ε*^*value*^+*ε*^*candidates*^)-differential privacy.)

#### *Proof*

*IPA* consists of four parts: (1) determining the suppression threshold, (2) adding noisy records, (3) choosing an informative value, and (4) choosing a node. We showed that each operation is differentially private on its own. As these operations run on the same dataset, based on Theorem 3, *IPA* achieves (*ε*^*suppression*^+*ε*^*insertion*^+*ε*^*value*^+*ε*^*candidates*^)-differential privacy. □

## Results and discussion

In this section, we present the experimental evaluation of *IPA* with respect to the utility of the output data and real-world analyses. For this evaluation, we use the NPS (National Patients Sample) dataset from HIRA (Health Insurance Review and Assessment which is a service in Korea) [20]. The NPS dataset consists of EHRs(Electronic Health Records) sampled from 3% sampled Korean people, in 2011. We analyze 1,361,000 records with 6 attributes: *Age, Sex, Length of stay in hospital, Location Surgery status,* and *Disease*. We consider the disease attribute as the informative attribute.

### Data utility

**Fig. 6:**
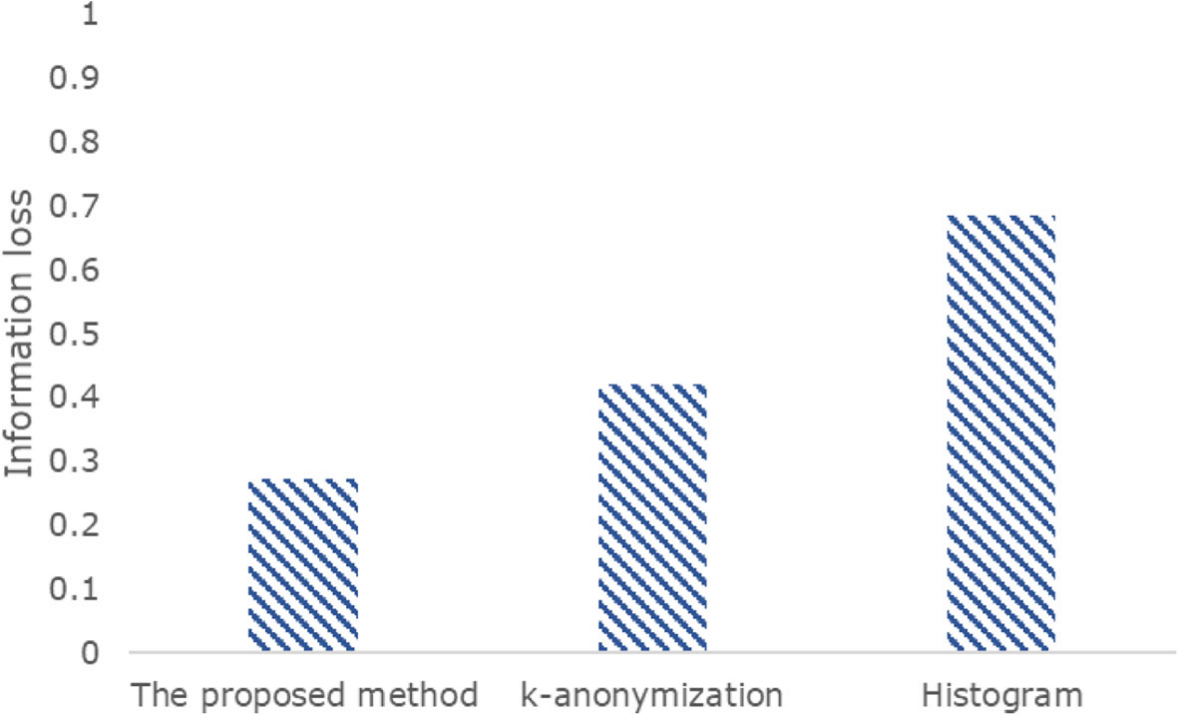
Comparison of the proposed and previous methods in terms of information loss

We measure the amount of distortion in the anonymized dataset in comparison with its raw version. We compare the proposed method with *k*-anonymization [17] and differentially private histogram methods [10]. In medical privacy settings, epsilon is typically set as 0.1-2 [14, 21, 22]. According to previous studies, 10-anonymity can be achieved when epsilon is equal to 1 [23]. Therefore, we set the parameter values as *ε* = 1 and *k* = 10. Figure 6 illustrates the information loss of anonymized datasets, where *ε* is 1 and *ε*^*suppression*^, *ε*^*insertion*^, *ε*^*value*^, and *ε*^*candidates*^ are 0.1, 0.3, 0.3, and 0.3, respectively. The information loss of *IPA*, *k*-anonymization, and the histogram are 0.28, 0.43, and 0.69, respectively, as shown in the figure. For each experiment, we executed 10 runs and averaged the results of all the runs. *IPA* achieves lower information loss than the other methods, while guaranteeing more rigorous privacy.

**Fig. 7:**
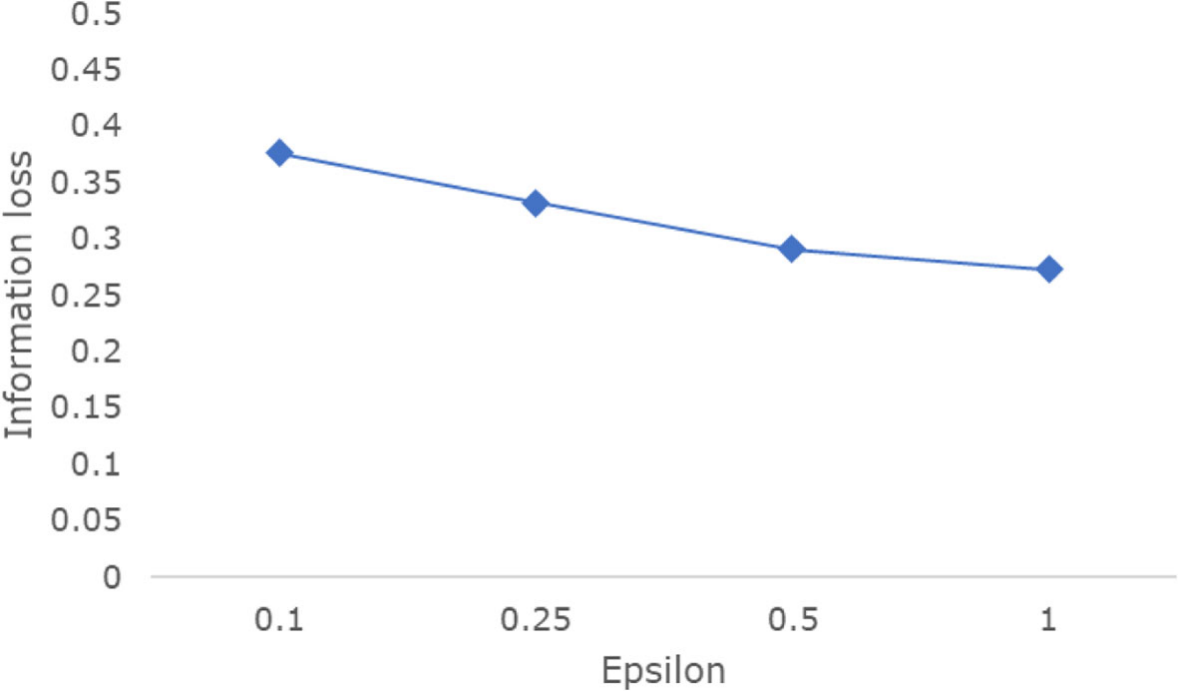
Information loss with varying *ε*

**Fig. 8:**
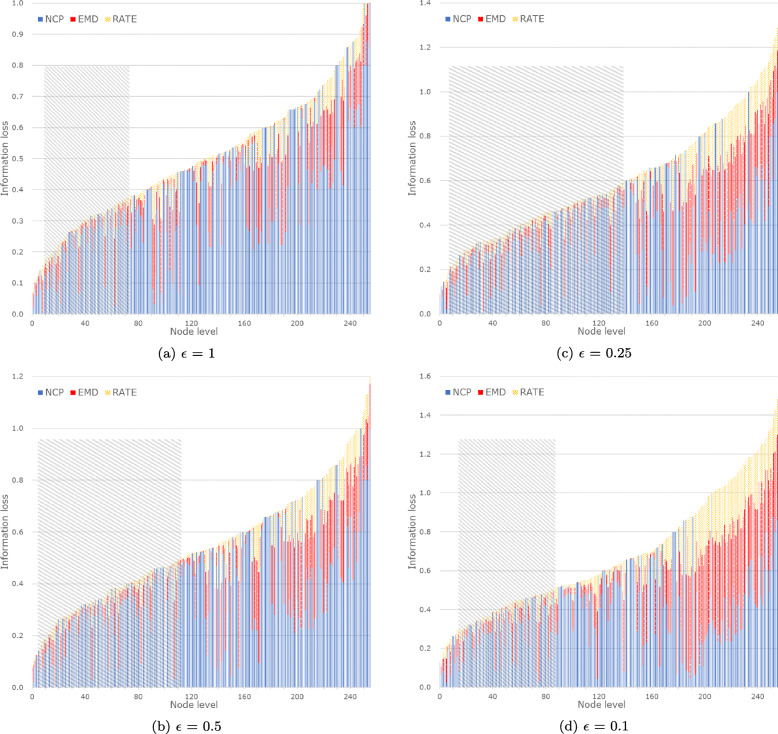
Information loss of candidate nodes

**Fig. 9:**
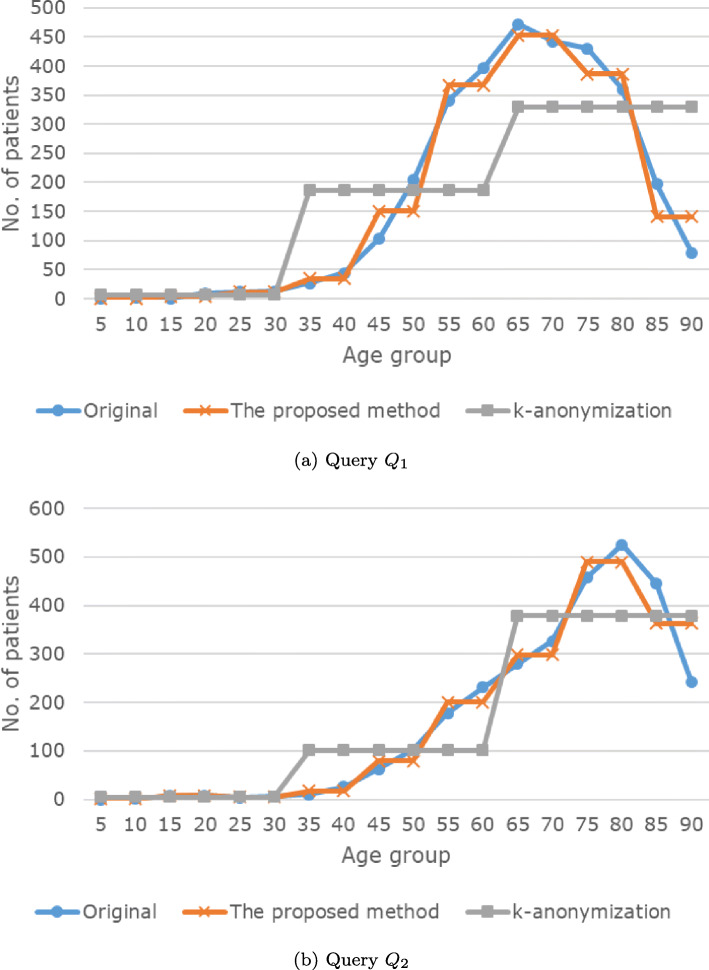
Results of the analysis queries

**Fig. 10:**
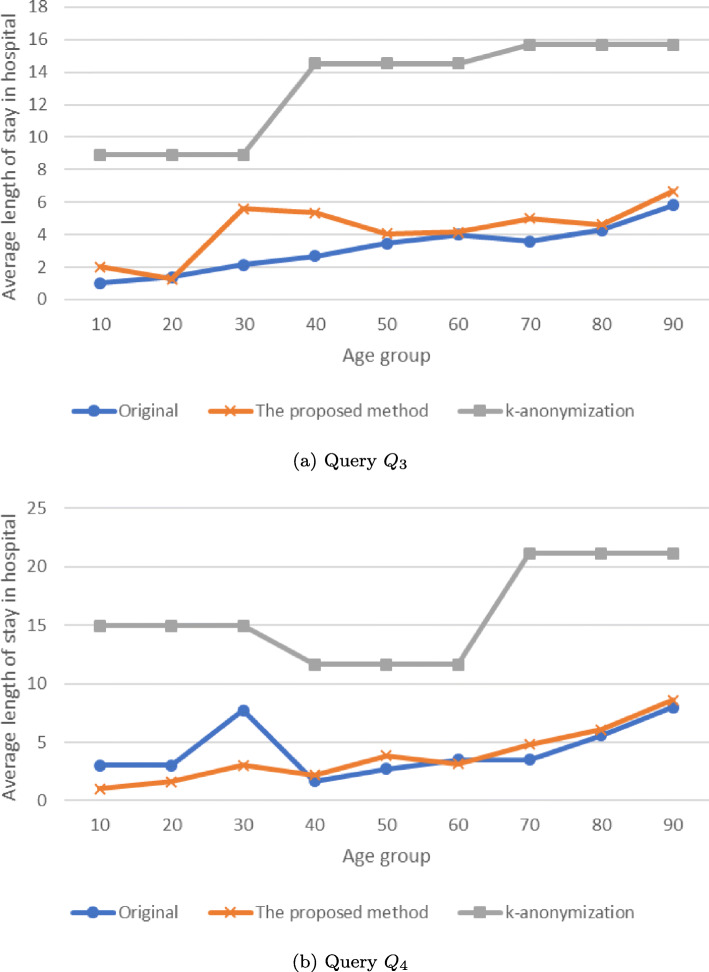
Results of the analysis queries

Figure 7 illustrates the information loss while varying the privacy budget *ε*. As expected, the information loss tends to decrease when *ε* increases. Figures 8, 9, and 10 provide the details. The proportions of *NCP*, *EMD*, and *Rate* in total information loss are represented by blue, red, and yellow lines, respectively. The x-axis denotes the node level in the hierarchical generalization lattice, and the area shaded with gray blocks represents the range from which experimental results are selected. For example, in Fig. 8a, the average information loss is 0.28, and the range is 0.16 to 0.38. As *ε* decreases, the proportions of *EMD* and *Rate* become larger than that of *NCP*, the gray block area increases, and the overall information loss increases. The range in Fig. 8d is narrower than that in Fig. 8c because lower level nodes are not selected by the score function as the overall information loss increases.

### Real-world analysis

We present a real-world analysis to illustrate the usefulness of *IPA*. We compare the results of *IPA* with those of the original dataset and of *k*-anonymity, using aggregation queries. The queries used for data analysis are as follows:

- ***Q***_**1**_: SELECT FLOOR(*Age*/5)*5 AS AgeGroup, COUNT(*) FROM *NPSdataset* WHERE *Sex*= ‘M’ and *Surgerystatus*= ‘N’ and *Disease*= ‘*stroke*’ GROUP BY FLOOR(*Age*/5)*5
- ***Q***_**2**_: SELECT FLOOR(*Age*/5)*5 AS AgeGroup, COUNT(*) FROM *NPSdataset* WHERE *Sex*= ‘F’ and *Surgerystatus*= ‘N’ and *Disease*= ‘*stroke*’ GROUP BY FLOOR(*Age*/5)*5
- ***Q***_**3**_: SELECT FLOOR(*Age*/5)*5 AS AgeGroup, AVG(*Lengthofstayinhospital*) AS Average length of stay in hospital FROM *NPSdataset* WHERE *Sex*= ‘M’ and *Surgerystatus*= ‘N’ and *Disease*= ‘*stroke*’ GROUP BY FLOOR(*Age*/5)*5
- ***Q***_**4**_: SELECT FLOOR(*Age*/5)*5 AS AgeGroup, AVG(*Lengthofstayinhospital*) AS Average length of stay in hospital FROM *NPSdataset* WHERE *Sex*= ‘F’ and *Surgerystatus*= ‘N’ and *Disease*= ‘*stroke*’ GROUP BY FLOOR(*Age*/5)*5

*Q*_1_ and *Q*_2_ represent the number of *stroke* patients for each age group (0-4, 5-9,...,86-90). *Q*_3_ and *Q*_4_ represent the average duration of stay in a hospital.

Figures 9 and 10 and Tables 9, 10, 11, and 12 present the results of the analysis queries. In Fig. 9, the x-axis represents the age group (which corresponds to the first projection column of *Q*_1_ and *Q*_2_) and the y-axis represents the number of *stroke* patients (which corresponds to the second projection column of *Q*_1_ and *Q*_2_). In Fig. 10, the x-axis represents the age group (which corresponds to the first projection column of *Q*_3_ and *Q*_4_) and the y-axis represents the average duration of stay in a hospital for *stroke* patients (which corresponds to the second projection column of *Q*_3_ and *Q*_4_). In each figure and table, the results of *IPA* are more similar to those of the original data, compared to the results of *k*-anonymity.

**Table 9:** Result of query *Q*_1_

Age group	Original	The proposed method	k-anonymization
5	1.0	1	6.3
10	2.0	1	6.3
15	1.0	4	6.3
20	9.0	4	6.3
25	12.0	12	6.3
30	13.0	12	6.3
35	27.0	34.5	186.2
40	44.0	34.5	186.2
45	104.0	150.5	186.2
50	205.0	150.5	186.2
55	341.0	367.0	186.2
60	396.0	367.0	186.2
65	472.0	452.5	329.8
70	442.0	452.5	329.8
75	430.0	386.5	329.8
80	360.0	386.5	329.8
85	197.0	141.5	329.8
90	78.0	141.5	329.8

**Table 10:** Result of query *Q*_2_

Age group	Original	The proposed method	k-anonymization
5	0.0	1.0	4.5
10	2.0	1.0	4.5
15	7.0	8.0	4.5
20	8.0	8.0	4.5
25	4.0	4.5	4.5
30	6.0	4.5	4.5
35	10.0	17.5	101.7
40	26.0	17.5	101.7
45	63.0	79.5	101.7
50	102.0	79.5	101.7
55	178.0	201.0	101.7
60	231.0	201.0	101.7
65	279.0	298.0	379.2
70	326.0	298.0	379.2
75	457.0	489.5	379.2
80	525.0	489.5	379.2
85	445.0	363.5	379.2
90	243.0	363.5	379.2

**Table 11:** Result of query *Q*_3_

Age group	Original	The proposed method	k-anonymization
10	1.0	2.2	8.9
20	1.4	1.4	8.9
30	2.1	5.7	8.9
40	2.7	5.5	14.5
50	3.4	4.2	14.5
60	4.0	4.1	14.5
70	3.6	4.8	15.7
80	4.3	4.5	15.7
90	5.8	6.5	15.7

**Table 12:** Result of query *Q*_4_

Age group	Original	The proposed method	k-anonymization
10	3.0	1.0	15.0
20	3.0	1.4	15.0
30	7.7	2.8	15.0
40	1.7	2.4	11.7
50	2.7	3.6	11.7
60	3.5	3.2	11.7
70	3.5	4.7	21.1
80	5.6	6.1	21.1
90	8.0	8.5	21.1

## Conclusions

Publishing anonymized microdata bestows additional flexibility to data recipients, as compared to providing sampled data or answers to specific queries. Considering this, we proposed a differentially private medical microdata releasing method that preserves measure attribute values; this proposed method is called *IPA*. To achieve this notion of privacy, we adopt differential privacy, which does not make any assumptions regarding the background knowledge of adversaries. To improve utility while preserving privacy, *IPA* employs three data perturbation methods: generalization, insertion, and suppression. *IPA* generalizes attribute values, except for measure attributes, to reduce the number of counterfeit records. Thereafter, it adds noisy records to achieve differential privacy; it also suppresses equivalent classes to avoid the addition of counterfeit records to empty equivalent classes. Through the results of our experiments, we demonstrated that *IPA* can reduce noise with an appropriate level of generalization. In addition, an experimental evaluation of a real-world data analysis proved that *IPA* can reduce information loss and also improve the utility of medical microdata published via differential private methods.

## Data Availability

The datasets generated and analyzed in the current study are available in the HIRA repository [20].

## Abbreviations

IPA: Informative attribute preserving anonymization
SSN: Social security number
NCP: Normalized certainty penalty
EMD: Earth mover’s distance
NPS: National patients sample
HIRA: Health insurance review and assessment
EHR: Electronic health record
